# Prognostic and Predictive Biomarkers in Head and Neck Squamous Cell Carcinoma Treated with Radiotherapy—A Systematic Review

**DOI:** 10.3390/biomedicines10123288

**Published:** 2022-12-19

**Authors:** Daniel H. Schanne, Alexander Koch, Olgun Elicin, Roland Giger, Michaela Medová, Yitzhak Zimmer, Daniel M. Aebersold

**Affiliations:** 1 Department of Radiation Oncology, Inselspital, Bern University Hospital and University of Bern, Freiburgstrasse 18, 3010 Bern, Switzerland; 2 Graduate School for Health Sciences, University of Bern, Mittelstrasse 43, 3012 Bern, Switzerland; 3 Department for BioMedical Research, University of Bern, Murtenstrasse 28, 3008 Bern, Switzerland; 4 Department of Otorhinolaryngology, Head and Neck Surgery, Inselspital, Bern University Hospital and University of Bern, Freiburgstrasse 18, 3010 Bern, Switzerland

**Keywords:** head and neck cancer, radiotherapy, biomarker, systematic review

## Abstract

*Background*: Radiotherapy is a mainstay in head and neck squamous cell carcinoma (HNSCC) treatment but is mostly applied without stratification by molecular diagnostics. Development of reliable biomarkers may have the potential to improve radiotherapy (RT) efficacy and reduce toxicity. We conducted a systematic review to summarize the field of biomarkers in HNSCC treated by RT. *Methods*: Pubmed and EMBASE were searched independently by two researchers following pre-defined inclusion and exclusion criteria. Z curves were generated to investigate publication bias. OncoKB was used for identification of druggable targets. *Results*: 134 manuscripts remained for data extraction. 12% of tumors were AJCC/UICC stage I–II and 82% were stage III–IV. The most common biomarkers were proteins (39%), DNA (14%) and mRNA (9%). Limiting analysis to prospective data and statistically significant results, we found three potentially druggable targets: ERCC2, PTCH1 and EGFR. Regarding data quality, AJCC/UICC stage was missing in 32% of manuscripts. 73% of studies were retrospective and only 7% were based on prospective randomized trials. Z-curves indicated the presence of publication bias. *Conclusion*: An abundance of potential biomarkers in HNSCC is available but data quality is limited by retrospective collection, lack of validation and publication bias. Improved study design and reporting quality might accelerate successful development of personalized treatments in HNSCC.

## 1. Introduction

Squamous cell carcinoma of the head and neck (HNSCC) remains the 6th most common cause of cancer worldwide [1]. In recent years, an increasing number of publications emerged, aiming to identify prognostic and predictive biomarkers but HNSCC is still a tumor entity characterized by a paucity of personalized treatments. The only targeted therapy approved in the USA and European Union in the curative treatment setting is cetuximab, an EGFR-binding antibody whose mechanism of action has been suggested to be driven in large parts by triggering immunologic anti-tumor reactions and not predominantly by inhibition of the EGFR pathway [2]. Therefore, in the curative setting, HNSCC is still mostly tackled by surgery and radiotherapy (RT; with or without concomitant systemic therapy) without a molecular stratification to choose an escalated or de-escalated strategy, as is common in other oncological diseases (reviewed in Kerr et al. [3], Deacon et al. [4]). Development of reliable and affordable biomarkers is therefore an eminent concern to improve HNSCC treatments.

A recent systematic review reported on clinical and biological biomarkers in HNSCC and summarized a total of 86 studies, regardless of applied therapy and treatment intent [5]. However, treatment approach might be an important consideration because it is inherently linked with differing patient characteristics and outcomes. Patients receiving either surgery or radiotherapy as primary treatment differ significantly in clinical features, especially tumor stage and anatomical location [6]. Additionally, RT is directly affected by multiple biological processes such as hypoxia or tumor stem cells. Oxygen is among the strongest modifiers of radiosensitivity, expressed in a tumor response approximately 2.5 to 3 times greater (oxygen enhancement ratio) if sufficient oxygen is present at the time of radiation or very shortly thereafter. This has initially been explained as the result of oxygen-induced fixation of radiation-induced DNA damage by free radicals [7]. However, recent research supports additional mechanisms, e.g., modulation of angiogenesis, cell stress responses and immune response (reviewed in Sørensen et al. [8]). Recently, hypoxia-based stratification of HNSCC patients treated with RT has entered clinical trials in an attempt to leverage this effect for clinical benefit [9]. Cancer stem cells (CSC) are another biological factor influencing RT outcome. It was shown that the presence of the putative CSC marker CD44 is associated with an increased risk of local recurrence in patients with laryngeal cancer [10]. This effect has been confirmed in two publications by the German Cancer Consortium Radiation Oncology Group in the setting of primary radio-chemotherapy in locally advanced HNSCC, and post-operative RT after surgical resection of HNSCC, respectively [11,12]. Similar to hypoxia, stratification of patients by stem cell status might be a promising strategy in HNSCC after confirmation of this approach in prospective clinical trials. Lastly, ionizing radiation itself also acts as a mutation-inducing agent and might affect biomarkers, e.g., by modulation of the tumor immune microenvironment [13,14].

In summary, RT is one of the main treatment modalities for HNSCC and its effects are directly affected by tumor biology. Our analysis therefore focuses exclusively on patients who received RT as part of their treatment with predominantly curative intent and strives to give an overview over the field of biomarkers in HNSCC from a quantitative and qualitative perspective. We omitted publications involving HPV as a prognostic biomarker and manuscripts on circulating HPV- and EBV-DNA as these topics have already been discussed in several systematic reviews and meta-analyses [15,16,17,18].

## 2. Materials and Methods

We searched Pubmed for available literature using the following search terms: ((head[Title/Abstract] OR neck[Title/Abstract] OR HNSCC[Title/Abstract]) AND (cancer[Title/Abstract] OR tumor[Title/Abstract]) AND (radiotherapy[Title/Abstract] OR radiation[Title/Abstract] OR irradiation[Title/Abstract]) AND (sequencing[Title/Abstract] OR proteomics[Title/Abstract] OR RNA[Title/Abstract] OR DNA[Title/Abstract] OR marker[Title/Abstract] OR serum[Title/Abstract] OR plasma[Title/Abstract] OR HPV[Title/Abstract]))) AND ((“2010/01/01”[Date—Publication]: “2022/03/01”[Date—Publication])) NOT (Review[PT] OR Meta-Analysis[PT] OR Systematic Review[PT]). To find additional literature, we also searched EMBASE using analogous search terms. Abstracts were independently screened by two physicians with experience in the field of HNSCC, radiation oncology and preclinical science. Inclusion criteria were: (1) HNSCC, (2) ≥10 patients, (3) association of biomarker with relevant oncological endpoint provided, (4a) all patients treated with RT, or (4b) separate outcome reported for the subgroup of patients treated with RT, (5) publication date between 1 January 2010 and 1 March 2022. Exclusion criteria were: (1) Reports solely focusing on circulating HPV or EBV DNA, and studies exclusively focusing on HPV-status as a biomarker because a plethora of recent literature, including systematic reviews and meta-analyses are already available on these topics [17,18,19]). (2) studies solely based on publicly available databases (TCGA, NCDB), (3) narrative and systematic reviews. In ambiguous cases, consensus between both researchers was established to include or exclude the respective study.

Data was then extracted from the full-text version of remaining entries. Studies including patients not treated with RT were handled as follows: the study was excluded if no separate outcome parameters were reported for the RT group (as per exclusion criteria). Patient and tumor characteristics were gathered separately for the RT group, if provided in the manuscript. Otherwise, characteristics for the whole cohort, including patients not treated with RT, were extracted. Whenever available, multivariable tests were preferred over univariable ones. All data analysis was performed in R (v4.1.0) with packages: dplyr, ggplot2. Z curves were generated following the method described by Brunner, Schimmack and Bartoš [20,21], employing the R package zcurve. Literature search, data extraction, analysis and reporting were performed in accordance with the PRISMA and COSMOS-E statements [22,23]. The review was registered in the International Prospective Register of Systematic Reviews (PROSPERO) with the ID CRD42022365752. For identification of potentially druggable targets, data from OncoKB was used, including items with a therapeutic level ≥ 3 (date of data request: 13 June 2022) [24]. Ongoing clinical trials for biomarkers were identified via https://clinicaltrials.gov (date of access: 27 November 2022).

## 3. Results

### 3.1. Literature Search and General Characteristics

**Figure 1:**
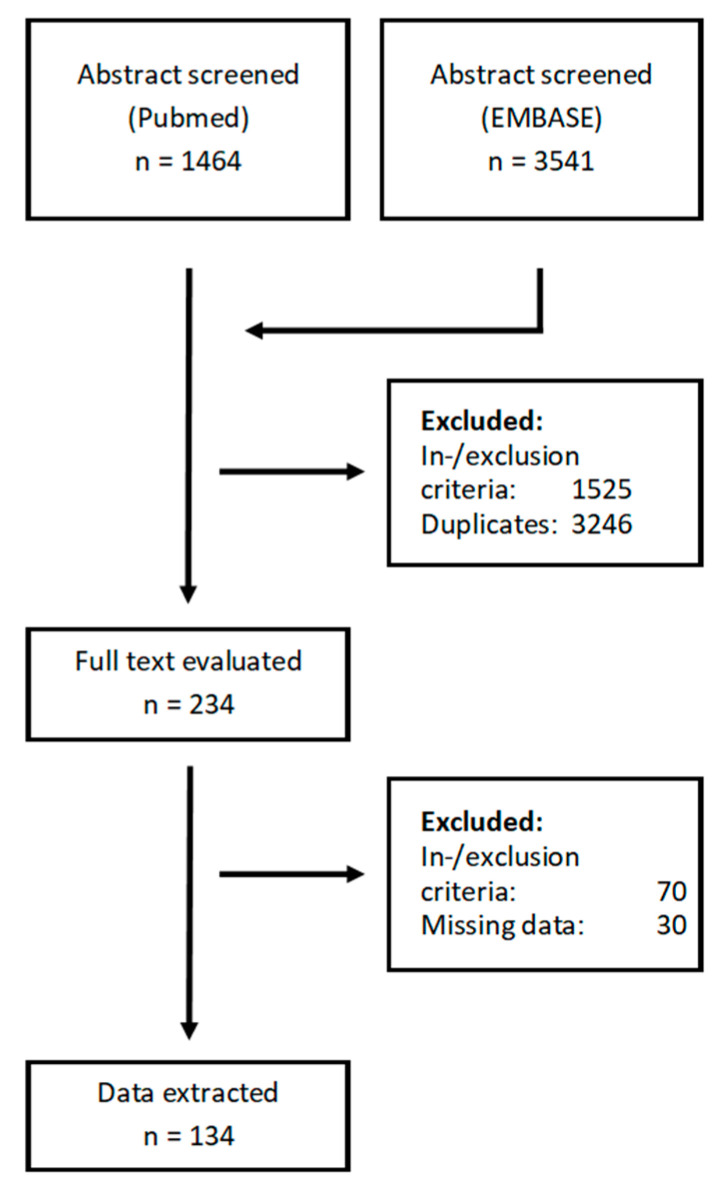
Screening process.

**Figure 2:**
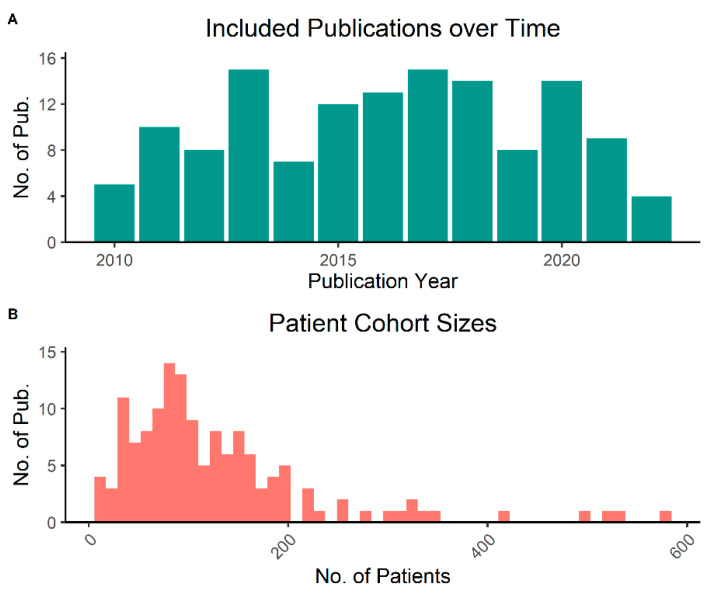
Number of included publications over the pre-defined time period (**A**). Histogram of the reported patient numbers that were included per study (**B**).

We screened 5005 publications from two databases and excluded 477 based on the inclusion and exclusion criteria. The 234 remaining studies were further evaluated for data quality and inclusion criteria in the full-text version, after which 134 manuscripts remained for data extraction (Figure 1). Median publication year of included studies was 2016 and there was no clearly discernible increase or decrease in the number of published manuscripts per year over time (Figure 2A). Studies had included a median of 100.5 patients but encompassed a large range spanning from 11 to 578 (Figure 2B).

### 3.2. Data Quality and Missingness

A relevant part of extracted variables was incompletely documented or could not be determined unequivocally from the description in abstract or full text. In 8.2% of publications, outcomes were reported for a group of patients treated with RT but clinical data was only available for the cohort as a whole (i.e., including patients who did not receive RT). The version of the used TNM classification was not mentioned in 61% of studies and AJCC/UICC stage was missing in 32%. Sufficient information about RT dose was not provided in 28% of publications and there was no information on smoking and alcohol consumption in 54% and 80% of reports, respectively. HPV-status was fully or partially missing in 66% of publications, translating to 47% of included patients. Regarding statistical analysis, 34% of extracted correlations between biomarker and outcome were based only on univariable analyses. To address publication bias, we generated z-curves for the most commonly used endpoints overall survival (OS), progression-free survival (PFS) and locoregional control (LRC) (Figure S1). We could demonstrate a marked discrepancy between the observed discovery rate (ODR) and the expected discovery rate (EDR) for all endpoints, indicating the presence of publication bias. Moreover, z-curves for OS and PFS demonstrated a difference in density immediately around z-score 1.96 (corresponding to *p* = 0.05), arguing for an overabundance of just-significant compared to just-non-significant results.

### 3.3. General Description of Included Studies

**Figure 3:**
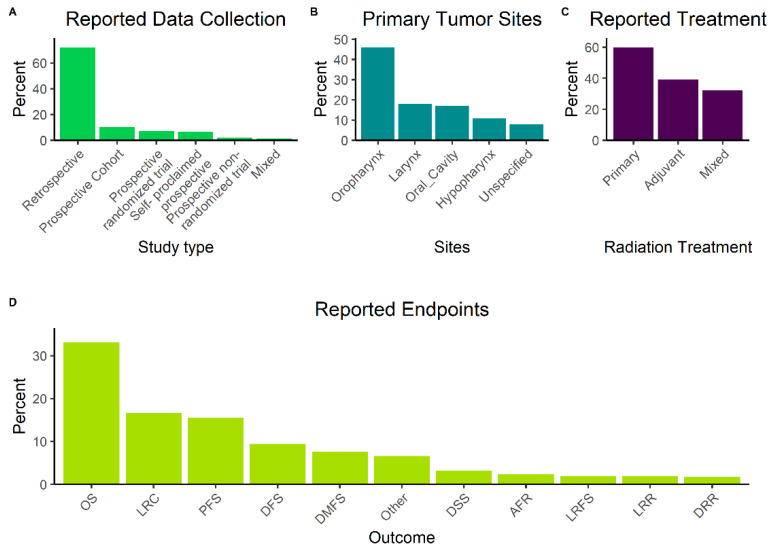
Method of data collection for included studies (**A**). Reported anatomical site of primary tumor (**B**). Type of radiotherapy applied to included patients (**C**). Reported Outcomes and endpoints (**D**). AFR—Any failure rate, DFS—Disease-free survival, DMFS—Distant metastasis- free survival, DRR—Distant recurrence rate, DSS—Disease-specific survival, LRC—Locoregional recurrence, LRFS—Local relapse-free survival, LRR—Locoregional recurrence, OS—Overall survival, PFS—Progression-free survival.

Among included studies, 7% were analyses of patient data from prospective randomized trials and another 10% from prospective, non-randomized interventional trials (Figure 3A). In 7% of publications, authors described the source of collected patient data as prospective but did not provide information about a potential study protocol or quality of patient follow-up and were hence classified as “self-proclaimed prospective”. Anatomically, the most commonly reported primary tumor site was oropharynx (46%), followed by larynx (18%), oral cavity (17%) and hypopharynx (11%) (Figure 3B). HPV-status was positive in 27% of all patients in studies with complete data, including two studies focusing only on HPV-positive cases. RT was described as the primary definitive treatment in 46%, and as adjuvant in 30% of publications; the remainder described mixed cohorts with both treatment intents. Analysis of TNM and AJCC/UICC stage was limited by the frequent unavailability of the used classification system’s version. Disregarding this restriction, only a minority of patients (12%) were early AJCC/UICC stage (I-II), whereas 81% were advanced stage (III-IV) and information was missing for 7%. Regarding RT, a multitude of fractionation schedules was employed, and reporting was frequently done in the format of a range. We therefore attempted to calculate the minimum applied equivalent dose 2 Gy (EQD2) that was described in each publication, resulting in a median of 66 Gy (range: 14 Gy–72 Gy). RT was mostly used in the primary or adjuvant setting but multiple publications also reported the use of both concepts in included patients (Figure 3C) Information about concomitant systemic therapy was missing in 16% of studies; the remaining publications reported application of a diverse range of regimens, including platinum compounds, nimorazole, mitomycin C, 5-fluorouracil, cetuximab, combinations thereof and others. Overall, in studies where information was available, 74% of patients received concomitant systemic therapy. Concerning endpoints, OS (33%) was the most frequently used, followed by LRC (17%), PFS (16%) and disease-free survival (DFS, 9%) (Figure 3D). A list of endpoint is provided in Abbreviations part.

### 3.4. Biomarkers

**Figure 4:**
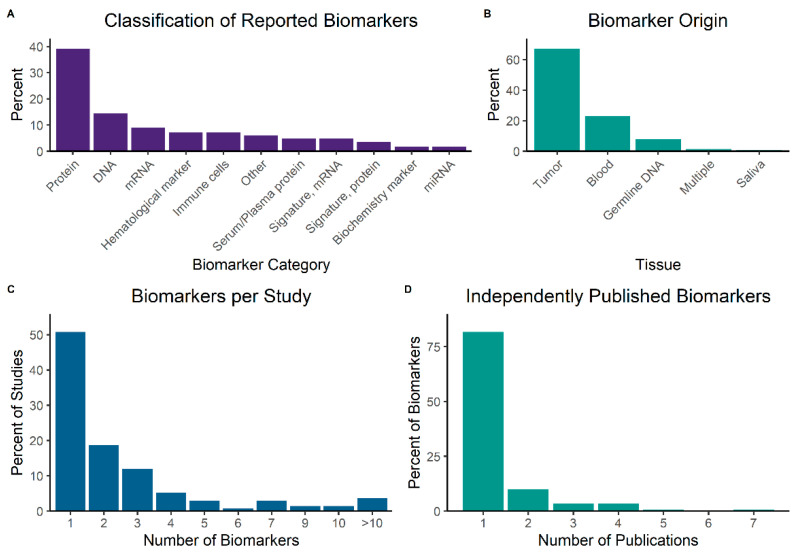
Distribution of biomarkers among categories (**A**). Tissue of origin for biomarkers (**B**). Number of assessed biomarkers per included study (**C**). Number of independent publications per biomarker (**D**).

We standardized biomarker names by replacing reported terms with HUGO symbols, where appropriate, and classified them in broad categories (Figure 4A). By far the largest category were proteins (39%), frequently detected by immunohistochemistry staining. Other common categories were DNA (14%), often assessed for mutations and copy number alterations by sequencing; mRNA (9%), commonly measured by reverse-transcription polymerase chain reaction (RT-PCR) or probes; and hematological markers (7%), subsuming measurements of red or white cells, or hemoglobin. Immune cells (7%) included tumor-infiltrating lymphocytes or other cells of immunological lineage infiltrating tumor tissue. Finally, we also assigned the categories serum/plasma proteins (e.g., albumin); and signatures, consisting of a combination of biomarkers. Regarding the tissue that biomarkers were extracted from, two-thirds (67%) of samples were biopsies or surgical specimens from tumors (Figure 4B). Only 23% were blood samples and a smaller share consisted of germline DNA (often from whole blood) and saliva. We further analyzed the number of biomarkers per included publication and found that the majority (51%) of papers dealt with one biomarker, with a marked fall-off at two (19%), three (12%) and four or more (18%) (Figure 4C). Lastly, we calculated the number of independent publications for each biomarker in our dataset and could demonstrate that the majority (82%) had only been assessed in one publication. Only 10% were described in two, and 8% in three or more studies (Figure 4D).

When assessing individual biomarkers that were reported to have a statistically significant association to outcomes and provided hazard ratios, the strongest positive predictors of local and locoregional tumor relapse was FGF2 (LRC, HR = 7.33, *p* < 0.001), detected in a retrospective study. However, when considering only prospectively collected data and multivariable tests, FANCC (LRR, HR = 6.6, CI [2.99–19.05], *p* = 0.0002), circulating tumor cells (CTC) (DFS, HR 4.3, CI [1.7–10.9], *p* = 0.002) and PTCH1 (LRR, HR = 5.98, CI [2.37–15.07], *p* = 0.0001) showed the strongest associations. Next, we correlated biomarkers found in our dataset with the OncoKB Precision Oncology Knowledge Base [17] and found three potential targets within therapeutic levels 1 to 3 (i.e., excluding only preclinical evidence, not considering disease-specificity or type of alteration): ERCC2, PTCH1 and EGFR (Table 1). A complete summary of statistically significant, multivariable tests in prospective studies, associated OncoKB data, and ongoing clinical trials involving respective markers (clinicaltrials.gov, as of 27 November 2022) is provided in Table 1, and a full list of included studies in Supplementary Table S1.

## 4. Discussion

Biomarkers have gained an ever-increasing role in the treatment of a wide spectrum of oncological diseases in recent years [3,4]. Treatment is now regularly guided by detection of gene expression or mutations, leading to an increasingly personalized approach. So far, HNSCC has not experienced the progress that other tumor entities have made in this regard. The aim of this work was therefore not only to summarize the available literature but also to identify potential obstacles that hinder progress in this field.

In our analysis, we could find a broad palette of biomarkers, ranging from well-known genes to lesser-studied targets. Among markers from higher-quality studies and analyses, we identified three targets that are potentially druggable according to OncoKB: (1) EGFR is a member of the ErbB family of receptors and binds to several of the epidermal growth factor (EGF) family of ligands. The EGFR pathway is involved in a multitude of cellular processes in cancer cells, most prominently the promotion of cell growth, division, migration and cell survival (reviewed in Normanno et al. [25]). EGFR has been studied extensively in the context of HNSCC and is an established clinical target with evidence of efficacy from randomized-controlled clinical trials [26]. (2) ERCC2, a DNA helicase that is involved in nucleotide excision repair [27]. Genetic alterations of this gene have been linked to increased chemotherapy-sensitivity, especially to cisplatin [28,29,30]. Interestingly, ERCC2 has also been implicated in a recent meta-analysis in higher tumor stage and grade, and a positive correlation with Ki-67 in HNSCC, suggesting a more aggressive tumor phenotype [31]. However, the hazard ratio (HR) of ERCC2 ranged between 0.42 and 2.07 in the three respective publications (Table S1) in our dataset, making a definitive interpretation difficult. (3) PTCH1, a member of the hedgehog signaling pathway with important roles in embryonic development and tumorigenesis (reviewed in Villavicencio et al. [32]). PTCH1 inactivation has been identified in early dysplastic lesions of the head-and-neck region [33] and is thought to be one of the main causes of nevoid basal cell carcinoma (BCC) syndrome, an autosomal dominant disease characterized by frequent BCC [34]. The relevant publication from our dataset assessed deletion and downregulation by methylation of PTCH1 and associated this with an increased risk for locoregional recurrence. The hedgehog pathway is druggable by sonidegib and vismodegib, both clinically approved for the treatment of BCC [35]. Another potentially druggable biomarker from a prospective cohort but without a reported HR in our analysis was the mutated variant of KRAS, G12C. A clinically approved treatment targeting this mutation in non-small cell lung cancer (NSCLC) [36] is available but unfortunately, the respective publication in our analysis did not indicate the specific KRAS mutation linked to the reported worse LRC. Prevalence of the G12C mutation has been estimated to be 0.24% in HNSCC [37]. A reliable statement about the mechanism of action (e.g., radio- or chemosensitization) of the four reported markers is not possible within the scope of this work, as none of the studies was designed to prove a causative, mechanistic relation between marker and an individual treatment.

Generally, prognostic markers are defined as characteristics that can be used to determine the probability of a pre-defined clinical event. On the other hand, predictive biomarkers allow to estimate an effect of exposure to an external factor, e.g., tumor response to a specific therapeutic intervention [38]. Considerable overlap exists between both categories and a distinction is only possible if there are two groups of patients, one with and one without the trait of interest. Additionally, a biomarker might be both prognostic and predictive. The most notable example for adoption of prognostic markers in HNSCC is usage of HPV-status for clinical and pathological staging [39,40]. There is, however, currently no established clinical consensus how the presence of HPV in HNSCC should affect therapeutic decisions as a predictive marker, but multiple clinical studies testing a de-escalation of systemic therapy or radiotherapy treatment have been published, recently, and further progress in this field is to be expected in the coming years [41,42,43]. Currently, no predictive markers are in routine clinical use for HNSCC with the exception of PD-L1 status and the combined positive score (CPS) before administration of pembrolizumab for palliative treatment [6]. Several studies are ongoing to establish predictive markers in the setting of radiotherapy in HNSCC, prominently the phase-III randomized DAHANCA-30 trial that tests administration of the radiosensitizer nimorazole, based on a hypoxia gene profile also included in our dataset (Table 1) [9,44]. Other significant efforts include development of the genomic-adjusted radiation dose (GARD) that was originally published in 2017 [45] and validated in HNSCC in a study in our dataset (HR = 4.9 for LRC, CI [1.97–12.3]) [46]. A recent pan-cancer validation study again found an overall effect on time to first recurrence but the subgroup of HNSCC demonstrated a small effect size and did not reach statistical significance (HR = 0.99, CI [0.96–1.01]) [47]. Among publications included in our study, a clear distinction between prognostic and predictive marker was often not possible because treatment was not uniformly applied to all patients, clinical data was missing, and most studies were not designed to demonstrate this difference.

When comparing our results with the recent systematic review of Budach et al., we identified 25 common biomarkers out of 246 in our dataset [5]. A certain overlap is expected between the two works because both are systematic reviews of biomarkers in HNSCC. However, our analysis focuses exclusively on patients receiving RT, covers a different time period and has set different inclusion criteria (e.g., minimum number of reported patients), thereby explaining the marked difference in results.

Our analysis has several strengths, including the systematic approach and a reasonably sized dataset of 134 included studies. We extracted not only outcomes but also quantifiable results such as hazard ratios and *p*-values, allowing some relevant analyses, e.g., of publication bias. One weakness of our study originates in the quality of data that is available in the literature. Our results show that more than 70% of included publications had been performed in a retrospective setting. While it is to be expected that hypothesis-generating studies are often done post-hoc, the inevitably introduced selection bias and lack of statistical power make follow-up studies necessary to confirm initial results. However, another finding was the lack of confirmatory and validation studies. While some included manuscripts had internal validation cohorts or were follow-up studies of earlier results, the majority of them reported isolated findings, leading to a large number of unvalidated hypotheses. This might in part be caused by publications not encompassed by our search terms but adding to this data, we found indications for marked publication bias in included studies in all three analyzed endpoints. In recent years, some authors have described a “reproducibility crisis” in various branches of scientific work with a large swath of results not being able to be replicated by independent research groups [48,49,50]. With the presented data in mind, it seems conceivable that the field of biomarkers in HNSCC might be at risk to be affected by this phenomenon.

In addition, another problem of our analysis was the unstructured reporting of included publications. Data was frequently categorized in arbitrary groups (e.g., different lumping of T or N stages) or only partially reported. In other cases, vital information was simply missing, such as the AJCC/UICC staging version, which has changed multiple times in recent decades with significant modifications over time, making a comprehensive comparison of included patient cohorts nearly impossible. Furthermore, the most frequently reported outcome was OS, which, as a composite endpoint, might not be appropriate for biomarkers if tumor response to a local or regional therapeutic regimen is to be measured. HNSCC patients regularly suffer from numerous comorbidities and severe treatment toxicity [51], acting as a competing risk for OS. Consequently, it has been reported in HNSCC that LRC does not consistently translate into OS [52].

Despite likely being the most widely-studied molecular marker in HNSCC, we omitted publications focusing on HPV as a prognostic marker for this work. HPV status has been of increasing importance in the clinical setting since the seminal analysis of the RTOG 0129 cohort [53] and has been introduced into the 8th UICC/AJCC staging system. Due to its preeminent stance among prognostic HNSCC markers, an abundance of publications on this topic is available, which has already spurred multiple systematic reviews and meta-analyses [15,16]. Including this large number of publications would have gone beyond the scope of this work. On similar grounds, we also excluded reports solely studying circulating HPV- or EBV-DNA, as these have been the subject of recent systematic reviews [17,18,19]. Of note, publications included in this review did contain HPV-positive as well as HPV-negative cases, but results were generally not stratified by HPV-status. The differing biology of tumors depending on HPV infection or, alternatively, exposure to other carcinogens might lead to divergent profiles of prognostic and predictive biomarkers, but data quality was not sufficient to discern this potential effect in our work.

## 5. Conclusions

In conclusion, the field of prognostic and predictive biomarkers in HNSCC shows a dynamic evolution during recent years and has the potential to significantly impact radiotherapy outcomes by reducing toxicity (e.g., via dose and volume de-escalation) and increasing tumor control (e.g., by exploiting radiosensitizing molecular alterations or increasing radiotherapy dose). However, considering the large number of published exploratory investigations, there is a lack of validation studies and prospective clinical trials to generate reliable, high-quality data to translate these findings into clinical practice. To achieve this, future studies should not only focus on the discovery of a marker or effect but validation in an independent patient cohort, ideally with pre-planned and prospectively collected data from multiple institutions. This will require intensive collaboration between pre-clinical scientists and physicians, beginning from the process of defining a marker of interest up to testing promising candidates in clinical trials. Relevant endpoints should be chosen for these studies, depending on the investigated disease, test or treatment. Additionally, to allow comparison of independently published manuscripts, stringent reporting standards should be maintained, as outlined, for example in the REMARK [54] guidelines. Lastly, the publication of results that are negative, but obtained from high-quality studies, would avoid unnecessary duplication of work and thereby enable a more efficient use of resources.

## Figures and Tables

**Table 1:** Biomarkers with statistically significant association to outcomes in multivariate analysis, OncoKB data and currently ongoing trials (clinicaltrials.gov identifier).

**Biomarker Category**	**Biomarker Name**	**PMID**	**Total Number of Patients**	**Outcome Category**	**Outcome Measure**	**Outcome Value**	**95% CI Lower**	**95% CI Upper**	**Outcome** ***p*-Value**	**OncoKB Drug (>=Level 3)**	**Ongoing Trials as of November 2022 (clinicaltrials.gov)**
Biochemistry marker											
	Beta-carotene	20358469	29	PFS	Hazard Ratio (HR)	0.30	0.09	0.96	0.04		
	Lutein	20358469	29	PFS	HR	0.21	0.05	0.92	0.04		
CTCs											
	Circulating tumor cells (CTCs)	25057171	144	DFS	HR	4.30	1.70	10.90	0.002		NCT05008796, NCT03926468
	CTCs	25057171	144	OS	HR	2.70	1.20	6.30	0.02		
DNA											
	CTLA4	22076708	531	OS	aHR (additive model)	1.32	1.08	1.62	0.01		
	CTLA4	22076708	531	OS	Global Wald test (co-dominant model)				0.02		
	Epigenetic Age Acceleration (EAA; end of RT)	33882281	146	OS	HR	1.33	1.15	1.62	<0.001		
	EAA (end of RT)	33882281	146	PFS	HR	1.32	1.16	1.54	<0.001		
	EAA (pre-RT)	33882281	146	PFS	HR	1.13	1.03	1.24	0.01		
	EAA (6 mo. post-RT)	33882281	146	PFS	HR	1.08	1.02	1.14	0.03		
	EAA (12 mo. post-RT)	33882281	146	OS	HR	1.15	1.01	1.33	0.04		
	ERCC1	22076708	531	DFS	Global Wald test (co-dominant model)				0.03		NCT02128906
	ERCC2	21890746	275	OS	HR	1.66	1.15	2.40	<0.01	Cisplatin	
	TNF	29802455	62	OS	HR	2.14	1.12	4.08	0.021		
	TP53	22076708	531	DFS	aHR (additive model)	1.28	1.02	1.60	0.03		NCT02734537
	XRCC1	22076708	531	OS	aHR (additive model)	1.28	1.05	1.57	0.02		
	XRCC1	22076708	531	OS	Global Wald test (co-dominant model)				0.03		
DNA/Epigenetic											
	FANCC	23482805	84	LRR	HR	6.60	2.48	17.57	0.0002		
	PTCH1	23482805	84	LRR	HR	5.98	2.37	15.07	0.0001	Sonidegib, Vismodegib	
Immune cells											
	TIL	33753155	39	LRC	HR	0.31	0.11	0.83	0.02		NCT05541016
miRNA											
	MIR15A	32266559	34	LPFS	HR	0.10	0.004	0.91	0.04		
mRNA											
	ATG12	34904929	103	LRC	Log-rank	-			0.03		
	ATG12	34904929	103	LC	Log-rank	-			0.04		
	SLC3A2	30993218	92	OS	Concordance index	0.65	0.57	0.73	0.01		
Protein											
	CXCR4	26374452	233	DMFS	HR	1.01	1.00	1.01	0.04		NCT03784066
	DNMT1	21284050	95	DFS	HR	2.55	1.32	4.90	0.01		
	EGFR	19733016	148	LRC	HR	1.35	1.00	1.82	0.004	Afatinib, Dacomitinib, Erlotinib, Erlotinib + Ramucirumab, Gefitinib, Osimertinib, Amivantamab, Mobocertinib, Erlotinib, Patritumab Deruxtecan, CLN-081, Poziotinib	NCT04456322
	ERCC1	24064970	90	PFS	HR	3.00	1.20	7.80	0.02		
	GLI2	27918595	36	OS	HR	0.40	0.16	0.95	0.03		
	IL6	21284050	95	DFS	HR	2.00	1.06	3.73	0.03		NCT03343236
	NME1	19733016	148	LRC	HR	1.65	1.05	2.59	0.01		
	p-STAT3	21284050	95	DFS	HR	2.16	1.26	3.72	0.01		
	PTEN	22413021	147	LRC	HR	2.84	1.38	5.80	0.004		NCT05172245
	SERPINE1	26359694	190	PFS	HR	1.92	1.03	3.59	0.04		
Serum/Plasma protein											
	CYFRA 21-1	28604997	185	OS	HR	2.33	1.14	4.73	0.02		
	CYFRA 21-1	28604997	185	DFS	HR	2.25	1.13	4.46	0.02		
	CXCL8	22383739	498	OS	HR	1.55			0.01		
	VEGFA	29658000	86	AFR	HR	0.71	0.55	0.91	0.01		
Signature, mRNA											
	15-gene hypoxia signature	21846821	323	LRC	HR	1.41	1.03	1.94	<0.05		NCT02661152, NCT02976051, NCT03865277, NCT01212354, NCT02352792, NCT00568490, NCT03513042, NCT04724096, NCT03323463

## Data Availability

Not applicable.

## Supplementary Materials

The following supporting information can be downloaded at: https://www.mdpi.com/article/10.3390/biomedicines10123288/s1.

## Abbreviations

AFR	Any failure rate
CSS	Cancer-specific survival
DFFS	Distant failure-free survival
DFS	Disease-free survival
DMFS	Distant metastasis-free survival
DRR	Distant recurrence rate
DSM	Disease-specific mortality
DSS	Disease-specific survival
EFS	Event-free survival
FFDM	Freedom from distant metastasis
FFR	Freedom from relapse
FFS	Failure-free survival
GOF	Goodness of fit
GOF	Goodness of fit
LC	Local control
LF	Local failure
LFFS	Local failure-free survival
LPFS	Local progression-free survival
LR	Local recurrence
LRC	Locoregional recurrence
LRF	Locoregional failure
LRFS	Local relapse-free survival
LRR	Locoregional recurrence
OS	Overall survival
PFS	Progression-free survival
RFS	Recurrence-free survival
RRFS	Regional recurrence-free survival
