# Supplementary figures and images for: Prognostic and Predictive Biomarkers in Head and Neck Squamous Cell Carcinoma Treated with Radiotherapy—A Systematic Review
Source: Biomedicines. 2022 Dec 19;10(12):3288. doi: 10.3390/biomedicines10123288 (PMC9775486; doi:10.3390/biomedicines10123288)

A Overall Survival

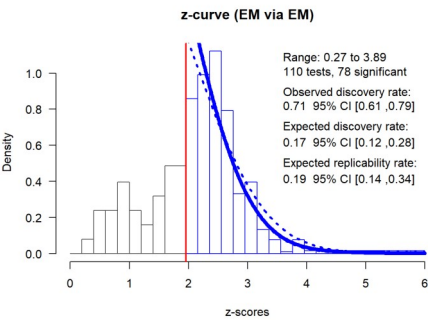

B Progression-free Survival

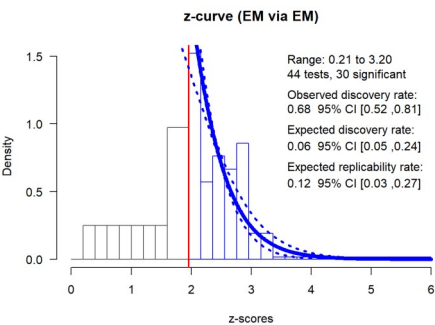

C Locoregional Control

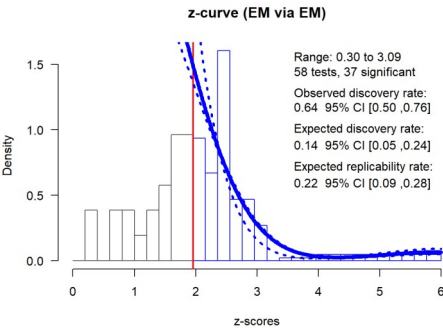

Supplement: Supplementary file 1 [file biomedicines-10-03288-s001.zip › Figure S1.pdf]
